# Dataset on the fear, preventive behaviour and anxiety disorder during the COVID-19 pandemic in Khyber Pakhtunkhwa, Pakistan

**DOI:** 10.1016/j.dib.2020.106579

**Published:** 2020-11-24

**Authors:** Waheed Ahmad Qureshi, Muhammad Saud, Qaisar Khalid Mahmood

**Affiliations:** a Department of Sociology, Anadolu Üniversitesi, Eskişehir, Turkey; b Department of Sociology, Faculty of Social and Political Sciences, Universitas Airlangga, Indonesia; c Department of Sociology, International Islamic University, Islamabad, Pakistan

**Keywords:** COVID-19, Mental health, Anxiety, Pandemic, Fear, Preventive behaviour, Pakistan

## Abstract

The outbreak of COVID-19 has raised numerous challenges to the present world. Due to the rapid spread of coronavirus, the high death toll, and enforcement of lock-down, people around the globe have faced fearful situations. Researches have also shown that a massive increase in psychological and mental health disorders is reported during the ongoing pandemic. The present cross-sectional data reflects the condition of the coronavirus fear, mental health, preventive behaviour, and anxiety disorder among the people of Khyber Pakhtunkhwa (KPK), Pakistan. At the time of data collection, the condition of lockdown and mobility restrictions were imposed by the provincial government due to which manual/physical collection of data was not possible. An online survey was designed using Google form facility to gather data from the respondents. After getting confirmation from pilot testing, the survey link was distributed through various online platforms including social media. Besides utilizing personal contact points in the KPK, social applications like Facebook, WhatsApp, and LinkedIn were also used for the dissemination of the survey. A total number of 501 respondents have furnished their responses and the survey was completed in a short period of time and the data were analyzed using SPSS. This data may be of great interest to researchers, policymakers, research organizations, social and mental health practitioners who wish to explore other dimensions of fear and anxiety among the masses caused by an ongoing pandemic (COVID-19).

**Specifications Table**

**Table:** 

Subject:	Mental Health; Social Sciences
Specific subject area:	Fear of COVID-19; Preventive Behaviour; Mental Well-being and Anxiety Disorder
Type of data:	Excel file Table
How data were acquired?	To collect data, an online questionnaire was developed by incorporating Fear of Corona Virus-19 Scale (FCV19S), Generalized Anxiety Disorder (GAD) Scale and Positive Mental Health Scale (PMHS). The facility of Urdu language translation was also provided in the questionnaire for the convenience of the respondents. The questionnaire is available online and can be accessed through the following link: https://forms.gle/s9NxJTbPCfeyajcM6
Data format:	Raw Filtered Analyzed
Parameters for data collection:	Respondents from three categories of age (up to 25 years; 26 to 50 and 51 & above) participated in this survey research.
Description of data collection:	The Khyber Pakhtunkhwa (KPK), previously known as the North-West Frontier Province of Pakistan, is situated in the northwestern region of the country and shares an international border with a neighbouring state of Afghanistan. It has a long history of tribal rivalries and conflicts which resulted in various social, psychological and security issues. Due to the war in Afghanistan, KPK also hosts one of the largest refugee populations in South Asia. The occurrence of above said events and unrest in the region have apparently contributed towards an increase of fear, anxiety, and depression among its residents. In the first half of 2020, the presence of COVID-19 was detected in Pakistan and a rise in positive cases was observed. In order to control the spread of the pandemic, the government of Pakistan has decided to impose lockdown restrictions. The KPK was the province where not only the rapid rise in the cases was found but also a high death toll reported due to COVID-19. Keeping in view this fearful situation and considering the timings appropriate, it was decided to conduct a research study there. Due to the rapid spread of COVID-19 in the country, restricted measures were imposed by the KPK (provincial) government from 25 March 2020, and we have collected data using an online cross-sectional survey during the lock-down period (May 10–23, 2020). The respondents were contacted through various social media platforms – Whatsapp, Facebook, and LinkedIn. Through a cover letter, the survey objectives were properly communicated to the respondents and their informed consent was obtained electronically. In order to ensure the confidentiality and anonymity of respondents’ information, the condition of contact sharing was eliminated from the survey. The survey link was also circulated through professional contacts in the academic and local organizations of the KPK. The faculty members serving in various provincial universities and colleges were requested for the dissemination of the survey link among their students, friends, and colleagues. The Urdu translation of all the questions was provided in the survey for respondents’ convenience. All the questions in the survey were marked with an asterisk and no survey response could be sent while leaving any question unanswered. A total of 501 respondents participated and no incentive was offered to them for their participation in this survey.
Data source location:	Institution: International Islamic University (IIU) City/Town/Region: Islamabad Country: Pakistan
Data accessibility:	This data is uploaded and published by Mendeley Name of Repository: Mendeley Data Data identification number: 10.17632/nzcrfhgfh4.2 Direct URL to data: https://data.mendeley.com/datasets/nzcrfhgfh4/2
Related research article:	Mahmood, Q.K., Jafree, S.R. & Qureshi, W.A. The Psychometric Validation of FCV19S in Urdu and Socio-Demographic Association with Fear in the People of the Khyber Pakhtunkhwa (KPK) Province in Pakistan. Int J Ment Health Addiction (2020). https://doi.org/10.1007/s11469–020–00371–4

**Value of the Data**  

• At the time of data collection, the KPK province was facing lock-down and the distress among the people caused by COVID-19 was supposed to be high. The presented data may help researchers and public/mental health practitioners to get a better insight of the relationship between the prevalence of the COVID-19 fear among the masses and their common precautionary measures practiced during the time of pandemic in KPK, Pakistan.
• Social as well as public/mental health researchers can be benefited because they explore how levels of COVID-19 fear vary among different socio-demographic groups and disadvantaged populations. These data can also be equally beneficial to the policymakers and governmental bodies for making better strategies during COVID-19 and for future pandemics.
• These data may be utilized to evaluate the role of the positive mental health to cope with the fear of COVID-19 among the people who practiced isolation and social/physical distancing. Moreover, it can be used to assess the relationship between positive mental health and preventive behaviour of the respondents.
• As the data exhibits the attributes of positive and negative mental health and preventive behaviour of the people. The researchers and policymakers can get further insight to develop better knowledge and practices from this rapidly evolving situation.

## 1 Data Description

The data exhibited in this article were gathered during the COVID-19 pandemic in Khyber Pakhtunkhwa (KPK), Pakistan. The purpose of this data collection was to examine the experiences, anxiety, and preventive behaviour of the people while the COVID-19 crisis was at its peak in Pakistan. The data were collected during May 2020 and respondents from the KPK province have participated. The data represents very interesting characteristics of respondents based on gender, age, marital status, employment status, area of living, and education in KPK. The demographic of the participants can be found in Table 1 of our published research article [1]. Keeping in view the prevalence of lock-down conditions, the survey for data collection was distributed online using social media platforms like Facebook, WhatsApp, and LinkedIn. We have also contacted faculty of various universities and colleges in the KPK through email and requested them for the dissemination of survey links among their colleagues, friends, and students. Besides Pashto (local language), people of KPK have a good understanding of national language (Urdu). All the questions of the survey were reproduced in Urdu to ensure the comfortability of the respondents with the language. The respondents were supposed to respond to each question as they could not submit the survey form if they skip any of them. We did not collect contact details of the respondents to ensure the anonymized responses of the respondents.

**Table 1:** Preventive behaviour of the people during the COVID-19 pandemic.

Preventive Behaviour	Mean score
I regularly wash my hands for 20 s.	3.78
I wear mask when go outside.	4.27
I avoid handshake and physical gestures of greetings.	3.96
I maintain physical/social distance while meeting others.	3.93
I do not meet (or avoid) people who have cough or flue.	4.07
I do not touch my face without washing my hands.	3.55
When water is not available, I use sanitizer to clean my hands.	3.60
Mean score of respondents’ preventive practices.	3.87

The dataset demonstrates the fear, preventive behaviour, mental health, and anxiety disorder status of the respondents. A total of 501 responses were received and analyzed with the help of SPSS. The data in .xlsx form is also available online and can be accessed through the Mendeley dataset link https://data.mendeley.com/datasets/nzcrfhgfh4/2

### 1.1 Fear of the COVID-19 infection

A recently designed FCV-19S was used to examine the fear of COVID-19 infection among the people of KPK. This scale consists of 7-items (e.g., item 4, “I am afraid of losing my life because of corona virus-19″ and item 5 “When watching news and stories about corona virus-19 on social media, I become nervous or anxious”) measured on a five-item Likert scale with endpoints labelled ‘strongly agree’ and ‘strongly disagree’. Like its validation in other languages, the FCV-19S is validated in Urdu language [1].

### 1.2 Preventive behaviour towards COVID-19

Keeping in view the recommendations of the World Health Organization [2], seven statements were developed to measure the preventive behaviour of the respondents towards the pandemic (COVID-19). The items included: “I regularly wash my hands for 20 s”; “I wear a mask when I go outside”;) “I avoid shaking hands as a form of greeting”; “I maintain social/ physical distance while meeting others”; “I avoid people who have a cough or flu”; and “I do not touch my face, mouth, nose, or eyes without washing my hands” (Table 1).

### 1.3 Positive mental health (PMH)

Positive Mental Health Scale (PMH-scale) was originally developed and validated by [3]. We have used the same scale, but its items were rated on a 5-points Likert scale ranging from ‘strongly disagree’ to ‘strongly agree’. The scale consists of nine-item e.g. item 3, ‘All in all, I am satisfied with my life’; ‘ item 6, ‘I am in good physical and emotional condition’ and item 9, ‘I am a calm, balanced human being’ (Table 2).

**Table 2:** Scale items of positive mental health (PMH).

Mental Well-being	Mean score
I am often a carefree and good spirit.	3.55
I enjoy my life.	3.86
All in all, I am satisfied with my life.	3.95
In general, I am confident.	4.05
I manage well to fulfil my needs.	3.97
I am in good physical and emotional condition.	4.05
I feel that I am actually well equipped to deal with life and its activities.	3.70
Much of what I do brings me joy.	3.96
I am a calm, balanced human being.	3.90
Mean score of PMH	3.88

### 1.4 Public reflection on safety measures

The respondents’ attitudes were assessed towards the measures enforced by the provincial government to restrict the spread of COVID-19 infection and ensure public safety. The question related to satisfaction about academic activities was also asked from the respondents for which the mean value was calculated as 2.46. Again, the respondents responded to each item on a five-point scale that was labelled as ‘very dissatisfied’; ‘dis-satisfied’; ‘neutral’; ‘satisfied’ and ‘very satisfied’ (Table 3).

**Table 3:** Peoples’ satisfaction about measures taken by the provincial government ‘to control the spread of pandemic’, ‘to ensure family's safety’ and ‘education’.

Respondents’ opinion on public health measures	Mean score
Are you satisfied with the steps taken by the government authorities to control the spread of Coronavirus?	3.25
Are you satisfied with your family's safety from COVID-19?	3.52
Are you satisfied with your studies during the prevailing situation of COVID-19?	2.46
Mean satisfaction score.	3.08

### 1.5 Generalized anxiety disorder

Self-report based Generalized Anxiety Disorder (GAD) scale was developed by [4] and the same is used by us to assess the anxiety disorder among the respondents. There are seven items in this scale that also described several interesting diagnostic features of general anxiety – “anxious or on edge, feeling nervous and worrying too much about various things”. These items are rated on a four-point Likert scale with endpoints labelled ‘not at all’ to ‘almost every day’ (Table 4).

**Table 4:** Assessment of Anxiety among the respondents through Generalized Anxiety Disorder (GAD) Scale.

Prevalence of Anxiety (over the last two weeks)	Mean score
. . . . . feeling nervous, anxious, or on edge.	1.47
. . . . . not being able to stop or control worrying.	1.46
. . . . . worrying too much about different things.	1.57
. . . . . trouble relaxing.	1.54
. . . . . being so restless that it is hard to sit still.	1.37
. . . . . becoming easily annoyed or irritable.	1.51
. . . . . feeling afraid, as if something awful might happen.	1.41
Mean GAD score.	1.47

## 2 Experimental Design, Materials and Methods

The facility of google form was used to develop this cross-sectional survey for data collection. Before sharing the link with the public, we have ensured that the survey form is error-free and easily accessible to everyone. A total number of 501 respondents had furnished their responses and the process of data collection was completed in less than two-week time. The variable like gender, age, marital status, employment status, area of residence, and educational attainment of the residents of KPK province was included to assess their socio-demographic characteristics. This survey was distributed among the respondents in various ways. Social media platform was also utilized for this purpose and survey link was shared through WhatsApp, Facebook, and LinkedIn. The academic staff of KPK universities and colleges had played a vital role in the speedy collection of the data. Six themes were presented in this survey i.e. socio-demographic features, fear of COVID-19, preventive behavior, mental well-being, satisfaction against the government measures taken for the control of pandemic spread and prevalence of anxiety among the people of KPK. Before the launch of survey, a pilot survey (*n* = 27) was conducted and the results of pilot test showed that there were no further amendments needed. A coding frame was also developed to convert data into digitized form, later the converted data was exported to SPSS for statistical analysis.

## Ethics Statement

All the procedures carried out in this study were in line with the concerned research committee. The respondents in this research was participated voluntary and their informed consent was obtained before online submission of the survey form.

## CRediT Author Statement

**Waheed Ahamad Qureshi:** wrote the initial draft of the data description of the manuscript, **Muhammad Saud:** reviewed it and prepared the results while, **Qaisar Khalid Mahmood:** has collected the data from the field.

## Declaration of Competing Interest

The authors earnestly declare that they do not have any competing interests which could impact the research work produced in this article.
